# Association of psychosocial factors with all‐cause hospitalizations in patients with atrial fibrillation

**DOI:** 10.1002/clc.23503

**Published:** 2020-11-10

**Authors:** Pascal B. Meyre, Anne Springer, Stefanie Aeschbacher, Steffen Blum, Nicolas Rodondi, Juerg H. Beer, Marcello Di Valentino, Peter Ammann, Manuel Blum, Rebecca Mathys, Christine Meyer‐Zürn, Leo H. Bonati, Christian Sticherling, Matthias Schwenkglenks, Michael Kühne, David Conen, Stefan Osswald, Swiss‐AF investigators

**Affiliations:** ^1^ Division of Cardiology, Department of Medicine University Hospital Basel Basel Switzerland; ^2^ Cardiovascular Research Institute Basel University Hospital Basel Basel Switzerland; ^3^ Institute of Primary Health Care (BIHAM) University of Bern Bern Switzerland; ^4^ Department of General Internal Medicine, Inselspital Bern University Hospital, University of Bern Bern Switzerland; ^5^ Department of Medicine, Cantonal Hospital of Baden and Molecular Cardiology University Hospital of Zürich Zürich Switzerland; ^6^ Department of Cardiology Ospedale San Giovanni Bellinzona Switzerland; ^7^ Biomedical Sciences Università della Svizzera italiana Lugano Switzerland; ^8^ Division of Cardiology St. Gallen Switzerland; ^9^ Department of Neurology and Stroke Center University Hospital Basel, University of Basel Basel Switzerland; ^10^ Epidemiology, Biostatistics and Prevention Institute University of Zurich Zurich Switzerland; ^11^ Population Health Research Institute McMaster University Hamilton Ontario Canada

**Keywords:** atrial fibrillation, health perception, psychosocial factors, unplanned hospitalization

## Abstract

**Background:**

A high burden of cardiovascular comorbidities puts patients with atrial fibrillation (AF) at high risk for hospitalizations, but the role of other factors is less clear.

**Hypothesis:**

To determine the relationship between psychosocial factors and the risk of unplanned hospitalizations in AF patients.

**Methods:**

Prospective observational cohort study of 2378 patients aged 65 or older with previously diagnosed AF across 14 centers in Switzerland. Marital status and education level were defined as social factors, depression and health perception were psychological components. The pre‐defined outcome was unplanned all‐cause hospitalization.

**Results:**

During a median follow‐up of 2.0 years, a total of 1713 hospitalizations occurred in 37% of patients. Compared to patients who were married, adjusted rate ratios (aRR) for all‐cause hospitalizations were 1.28 (95% confidence interval [CI], 0.97‐1.69) for singles, 1.31 (95%CI, 1.06‐1.62) for divorced patients, and 1.02 (95%CI, 0.82‐1.25) for widowed patients. The aRRs for all‐cause hospitalizations across increasing quartiles of health perception were 1.0 (highest health perception), 1.15 (95%CI, 0.84‐1.59), 1.25 (95%CI, 1.03‐1.53), and 1.66 (95%CI, 1.34‐2.07). No different hospitalization rates were observed in patients with a secondary or primary or less education as compared to patients with a college degree (aRR, 1.06; 95%CI, 0.91‐1.23 and 1.05; 95%CI, 0.83‐1.33, respectively). Presence of depression was not associated with higher hospitalization rates (aRR, 0.94; 95%CI, 0.68‐1.29).

**Conclusions:**

The findings suggest that psychosocial factors, including marital status and health perception, are strongly associated with the occurrence of hospitalizations in AF patients. Targeted psychosocial support interventions may help to avoid unnecessary hospitalizations.

**Trial registration:**

ClinicalTrials.gov Identifier NCT02105844.

**Abbreviations**
AF: atrial fibrillation
BMI: body mass index
IQR: interquartile range
SD: standard deviation
TIA: transient ischemic attack
VAS: visual analogue scale

## 1 INTRODUCTION

Atrial fibrillation (AF) is expected to affect nearly 18 million Europeans in the future.^1^ Patients with AF have multiple comorbidities and a high risk of complications,^2^, ^3^, ^4^ which puts them at increased risk of being admitted to the hospital.^5^ Although many hospitalizations are likely triggered by medical conditions, nonmedical factors may also be crucial.

It is well‐established that social and psychological conditions (eg, marital status, education, mental health) play an important role in determining an individual's health.^6^ These psychosocial factors have been associated with the risk of cardiovascular adverse events,^7^, ^8^ and evidence suggests that the effects are comparable in strength to those associated with physical activity, smoking, or alcohol use.^9^, ^10^ Prior studies addressed the relationships of psychosocial risk factors with incident AF and heart failure hospitalizations.^11^, ^12^, ^13^ Among AF patients, those with a low social status, low education, or low household income had a higher risk of death as compared to individuals without such psychosocial constraints.^14^

However, only little is known whether psychosocial factors affect the risk of hospitalizations in AF patients. For instance, patients with low social support may be less able to cope with serious health conditions and life crises, which may increase their tendency to seek medical advice and hospital care. Given that hospitalizations are strong drivers of healthcare expenditures, more evidence on this topic may help to establish new preventive strategies. We therefore aimed to investigate the prevalence of psychosocial factors and their associations with all‐cause hospitalizations in a large cohort of well‐characterized patients with AF.

## 2 METHODS

### 2.1 Study Population

The Swiss Atrial Fibrillation Cohort (Swiss‐AF) is a large prospective cohort study of patients who had previously diagnosed AF enrolled across 14 centers in Switzerland. Details of the study design and first results have been published previously.^15^, ^16^ Patients were enrolled if they had documented AF and were aged 65 years or older. Exclusion criteria were short, reversible AF episodes (ie, AF occurring after cardiac surgery) or inability to give informed consent. The study protocol was approved by the local ethics committees, and written informed consent was obtained from all participants.

### 2.2 Assessments

Demographic and clinical information were collected using standardized case report forms and validated questionnaires. Yearly follow‐up visits were performed by local study personnel to collect patient characteristics, clinical measures and outcome events. Marital status and education level were social factors captured by the case report forms; depression and health perception were available psychological components. Participants were asked if they were married, single, divorced or widowed. Education level was evaluated using the sum of completed years at school, high school or college, and defined as primary or less (≤6 years), secondary (high school or similar: 6 to ≤12 years) and college or university (college or university degree: >12 years of education). Depression and depressive symptoms were measured using the Geriatric Depression Scale (GDS),^17^ with a total point score ranging from 0 to 15, and a total score of >5 points was used to indicate depression.^18^ Health perception was self‐assessed by patients indicating their current state of health using a visual analogue scale (VAS) ranging from 0 (worst) to 100 (best). The VAS used in this study was based on the EuroQol VAS and has been validated for AF patients.^19^, ^20^ For the purpose of the present analyses, we divided patients into quartiles of total VAS; the first quartile was defined as the reference (highest health perception).

### 2.3 Outcome

The outcome of this study was all‐cause hospitalization, defined as any unplanned admission leading to at least one overnight stay. Elective hospitalizations or emergency department evaluations were not counted. The occurrence of events was assessed at yearly follow‐up examinations through on‐site visit, phone call, or information gathered from the family physician.

### 2.4 Statistical analysis

Baseline characteristics are presented as means ± standard deviations (±SD) for continuous variables and as counts (percentages) for categorical variables. To account for the repeated occurrence of hospitalizations within patients, we used the total number of all‐cause hospitalizations as the primary outcome and applied negative binomial regression models to calculate rate ratios and 95% confidence intervals (CI). Models were adjusted for a predefined set of cardiovascular and noncardiovascular variables known to be associated with hospitalizations.^21^ These variables consisted of age, sex, body mass index (BMI), history of hypertension, diabetes, coronary heart disease, prior stroke or transient ischemic attack (TIA), heart failure, peripheral vascular disease, renal failure, cancer, and previous falls. We then constructed a combined multivariable model including all psychosocial factors and covariates to determine the strongest predictors for all‐cause hospitalizations.

In a next step, we conducted time‐to‐event analyses to find out how psychosocial factors influence the risk of first all‐cause hospitalization. We used Kaplan‐Meier methods to estimate the cumulative incidence of first all‐cause hospitalization across psychosocial factors and curves were compared by the log‐rank test. Incidence rates were calculated per 100 patient‐years of follow‐up. We constructed multivariable Cox proportional hazards models to test the association of psychosocial factors with the risk of first all‐cause hospitalization, adjusted for the same variable set as described above, and calculated the hazard ratios with corresponding 95% CIs. As indicated above, we also built a combined multivariable model including all psychosocial factors and covariates in a single model.

Multivariable and combined models included 2373 patients due to missing data of 5 patients (0.2%). All analyses were performed using Stata, version 13 (StataCorp. 2013. College Station, TX: StataCorp LP). A P value of <0.05 was defined as statistical significant.

## 3 RESULTS

From March 2014 through September 2017, a total of 2415 patients were enrolled into the study. Of those, 37 (1.5%) were excluded from the present analysis due to drop‐out, consent withdrawal or missing values regarding psychosocial factors. Thus, the analyses included a total of 2378 patients. Details regarding to patient selection are provided in the Figure S1 in the Supplement.

Baseline characteristics are shown in Table 1. Mean age of the participants was 73.2 (±SD, 8.4) years, and 647 (27.2%) were women. With regard to marital status, 1597 (67.2%) were married, 156 (6.6%) were single, 289 (12.2%) were divorced, and 336 (14.1%) were widowed. Two hundred and eighty‐one patients (11.8%) had primary or less education, 1181 (49.7) had secondary education, and 916 (38.5%) had a college or university degree. Depression was present in 112 (4.7%) patients and the median health perception was 75 (interquartile range [IQR], 60‐85).

**TABLE 1:** Characteristics of patients at baseline

Characteristics	Value, No. (%)
Patients, No.	2378
Age, mean (SD), y	73.2 ± 8.4
Female sex	647 (27.2)
Marital status	
Married	1597 (67.2)
Single	156 (6.6)
Divorced	289 (12.2)
Widowed	336 (14.1)
Education level	
Primary or less	281 (11.8)
Secondary	1181 (49.7)
College, or university	916 (38.5)
Depression or depressive symptoms	112 (4.7)
Health perception, median (IQR)	75 (60–85)
Atrial fibrillation type	
Paroxysmal	1067 (44.9)
Persistent	699 (29.4)
Permanent	612 (25.7)
Body mass index, median (IQR), kg/m^2^	27.0 (24.4‐30.3)
Medical history	
Hypertension	1651 (69.4)
Diabetes	405 (17.0)
Coronary artery disease	725 (30.5)
Stroke/TIA	474 (20.0)
Heart failure	616 (25.9)
Peripheral vascular disease	190 (8.0)
Bleeding	371 (15.6)
CHA_2_DS_2_‐VASc score, median (IQR)	3 (2–5)
Oral anticoagulation	2150 (90.4)
Vitamin K antagonist	939 (39.5)
Direct oral anticoagulants	1210 (50.9)
Antiplatelet therapy	470 (19.8)

Abbreviations: IQR, interquartile range; CHA_2_DS_2_‐VASc, congestive heart failure, hypertension, age 75 ≥ years (two points), diabetes, prior stroke or TIA or thromboembolism (two points), vascular disease, age 65 to 74 years, female sex; Bleeding, major bleeding or clinically relevant nonmajor bleeding.

**FIGURE 1:**
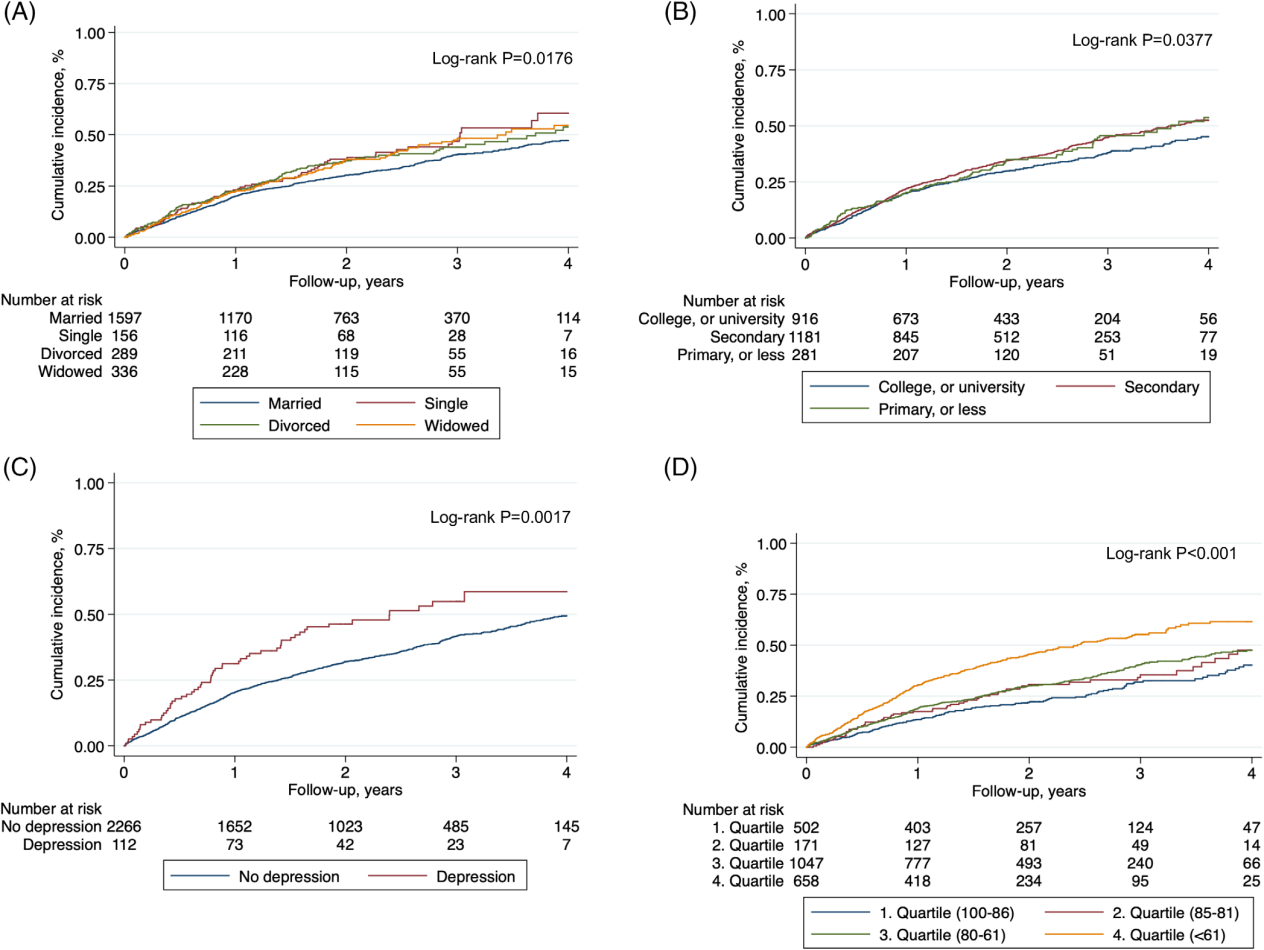
Cumulative Incidence of All‐Cause Hospitalization According to Psychosocial Factors. Cumulative incidence of all‐cause hospitalization according to marital status, A, education level, B, presence of depression, C, and quartiles of health perception, D. For health perception, the 1. quartile indicates health perception between 100 to 86; 2. quartile between 85 to 81; 3. quartile between 80 to 61; 4. quartile below 61

Over a median follow‐up of 2.0 years (IQR, 1.0‐3.0), there were a total of 1713 all‐cause hospitalizations. The cumulative incidences according to individual components of psychosocial factors are presented in the Figure 1. In multivariable analyses, patients who were divorced experienced higher rates of hospitalizations compared to those who were married (rate ratio [RR], 1.36; 95% CI, 1.10‐1.68; *P* = .005) (Table 2). This finding persisted in the combined model (RR; 1.31; 95% CI, 1.06‐1.62; *P* = .013). There was evidence that patients who reported lower health perception had a higher rate of hospitalization compared to patients with high health perception. In the multivariable model, the RRs for those in the second, third and highest quartiles of health perception, as compared to those in the in first quartile (reference), were 1.15 (95% CI, 0.84‐1.59; *P* = .38), 1.25 (95% CI, 1.03‐1.53; *P* = .026), and 1.68 (95% CI, 1.35‐2.08; *P* < .001), respectively (Table 2). We found similar findings in the combined model, with RRs of 1.15 (95% CI, 0.84‐1.59; *P* = .39), 1.25 (95% CI, 1.03‐1.53; *P* = .027), and 1.66 (95% CI, 1.34‐2.07; *P* < .001) across quartiles. There were no significant differences in hospitalization rates according to level of education or presence of depression, in multivariable and combined models.

**TABLE 2:** Relation between psychosocial factors and total all‐cause hospitalizations

	Total (first and recurrent) all‐cause hospitalizations							
Variables	Total no. of events^a^	No. at risk	Unadjusted RR (95% CI)	*P* value	Adjusted RR (95% CI)^b^	*P* value	Combined adjusted RR (95% CI^c^	*P* value
Marital status								
Married	1065	1597	1 [Reference]		1 [Reference]		1 [Reference]	
Single	135	156	1.30 (0.98‐1.72)	.070	1.28 (0.97–1.69)	.09	1.28 (0.97–1.69)	.08
Divorced	256	289	1.33 (1.07‐1.65)	.010	1.36 (1.10–1.68)	.005	1.31 (1.06–1.62)	.013
Widowed	257	336	1.15 (0.93‐1.41)	.19	1.03 (0.83‐1.27)	.80	1.02 (0.82–1.25)	.89
Education level								
College, or university	617	916	1 [Reference]		1 [Reference]		1 [Reference]	
Secondary	880	1181	1.11 (0.95‐1.29)	.20	1.05 (0.90‐1.22)	.51	1.06 (0.91–1.23)	.49
Primary or less	216	281	1.14 (0.90‐1.44)	.27	1.07 (0.84‐1.36)	.58	1.05 (0.83–1.33)	.68
Depression								
No	1606	2266	1 [Reference]		1 [Reference]		1 [Reference]	
Yes	107	112	1.35 (0.98‐1.85)	.07	1.08 (0.79‐1.49)	.61	0.94 (0.68–1.29)	.69
Health perception								
1. Quartile (100‐86)	231	502	1 [Reference]		1 [Reference]		1 [Reference]	
2. Quartile (85‐81)	93	171	1.18 (0.85‐1.64)	.32	1.15 (0.84–1.59)	.38	1.15 (0.84‐1.59)	.39
3. Quartile (80‐61)	691	1047	1.43 (1.17‐1.75)	<.001	1.25 (1.03–1.53)	.026	1.25 (1.03–1.53)	.027
4. Quartile (<61)	698	658	2.31 (1.87‐2.84)	<.001	1.68 (1.35–2.08)	<.001	1.66 (1.34–2.07)	<.001

*Note:* Data are presented as rate ratios (RR) with 95% confidence intervals (CI).

^a^ Total hospitalizations included first and recurrent events.

^b^ Models were adjusted for age, sex, body mass index, hypertension, diabetes, coronary heart disease, prior stroke/TIA, heart failure, peripheral vascular disease, renal failure, cancer, and previous falls.

^c^ Model was combined and adjusted for age, sex, body mass index, hypertension, diabetes, coronary heart disease, prior stroke/TIA, heart failure, peripheral vascular disease, renal failure, cancer, and previous falls.

During the same follow‐up period, there were 891 (37%) patients who experienced at least one hospitalization. Table S1 in the Supplement shows the incidence rates of first all‐cause hospitalization stratified by psychosocial factors. In multivariable analyses, patients who were single (hazard ratio [HR], 1.37; 95% CI, 1.06‐1.77; *P* = .015) or divorced (HR, 1.25; 95% CI, 1.02‐1.53, *P* = .030), had higher risk of first hospital admission compared to those who were married (Table S2). Similar associations were observed in the combined model, with HRs of 1.35 (95% CI, 1.05‐1.74; *P* = .021) and 1.23 (95% CI, 1.00‐1.50; *P* = .046), respectively. With regard to health perception, compared to patients who were in the first quartile (reference), the HRs of those who were in the second, third and fourth quartile were 1.22 (95% CI, 0.90‐1.65; *P* = .21), 1.13 (95% CI, 0.93‐1.38; *P* = .23) and 1.53 (95% CI, 1.24‐1.90; *P* < .001), in multivariable analyses (Table S2). We found similar findings in the combined model, with HRs of 1.22 (95% CI, 0.90‐1.65; *P* = .21), 1.13 (95% CI, 0.92‐1.38; *P* = .24) and 1.49 (95% CI, 1.21‐1.85; *P* < .001), respectively. Level of education and presence of depression were not associated with the risk of first hospitalization, in multivariable and combined models.

## 4 DISCUSSION

The present study investigated relationships between psychosocial factors and the risk of unplanned hospitalizations in patients with AF. Several important findings emerged. First, the rate of first and recurrent hospitalizations was high. Second, being divorced or having a low health perception was associated with a higher risk for unplanned hospitalizations. Third, depression and low education level were not associated with first or recurrent all‐cause hospitalizations.

Our study showed that patients who were single or divorced revealed a higher risk of hospitalizations relative to those who were married. Consistently, previous studies from nonAF populations showed higher hospitalization rates for unmarried compared to married individuals.^22^, ^23^ These findings are in line with the notion that compared to those living alone (single, divorced), patients who have close relationships to others can rely on better social support,^24^ while those with lacking support show increased needs for hospital care.^25^

Our results further indicated that the risk of hospitalizations was closely associated with the patients' subjective perception of health. Specifically, patients who felt in good health conditions were less likely to be admitted to the hospital. Evidence from studies of nonAF populations showed that patients who reported poor or fair health conditions exhibited an up to five times higher risk of hospitalization or death as compared to those reporting excellent or good health.^26^, ^27^ Previous studies suggested that self‐efficacy is a key predictor of heart failure hospitalization and all‐cause death.^28^ One may assume that social support and help of close others strengthen self‐efficacy beliefs, acting as a buffer of distress due to medical illness, which prevents patients with high social support from striving for hospital care. This view also corresponds to the high rates of hospitalization in unmarried patients observed in the present study. Moreover, low health perception has often been reported in AF populations.^29^

The clinical implication of our findings is that a better awareness of the patients' psychosocial conditions may help clinicians to intervene more sensitively and to be more responsive in offering specific support. Such interventions may include to improve the patient's social relations and to strengthen their self‐efficacy in face of illness, which may imply psychosocial counseling, self‐helping group assignment, or psychotherapy. To highlight this issue, future studies should include direct measures of social support. Also, effects of psychosocial treatment as complementary strategy of AF‐related medical treatment should be addressed.

### 4.1 Strengths and limitations

This is the first large cohort study of patients with AF evaluating the associations of psychosocial factors with hospitalizations. However, some limitations need to be discussed. First, the variables representing psychosocial factors were collected based on availability in the cohort data set. Additional factors, such as income or measures of social deprivation, may matter as well. Second, our results were derived from a national cohort of AF patients in Switzerland, a high‐income country with high medical standards, and the potential generalizability to other settings is unknown.

## 5 CONCLUSIONS

Our findings suggest that low social support is associated with an increased risk of unplanned hospitalizations in AF patients. The strongest factors associated with unplanned hospitalizations were marital status and health perception. Hence, comprehensive care with targeted psychosocial support interventions in addition to medical treatment may help to avoid unnecessary hospitalizations.

## CONFLICT OF INTEREST

Dr Bonati reports personal fees and nonfinancial support from Amgen, grants from AstraZeneca, personal fees and nonfinancial support from Bayer, personal fees from Bristol‐Myers Squibb, personal fees from Claret Medical, grants from Swiss National Science Foundation, grants from University of Basel, grants from Swiss Heart Foundation, outside the submitted work. Dr Sticherling has received speaker honoraria from Biosense Webster and Medtronic and research grants from Biosense Webster, Daiichi‐Sankyo, and Medtronic. Dr Schwenkglenks reports grants from Swiss National Science Foundation, during the conduct of the study; grants and personal fees from Amgen, grants from MSD, grants from Novartis, grants from Pfizer, grants from The Medicines Company, outside the submitted work. Dr Kühne reports personal fees from Bayer, grants from Bayer, personal fees from Pfizer‐BMS, personal fees from Daiichi‐Sankyo, personal fees from Böhringer‐Ingelheim, outside the submitted work. Dr Conen received consulting fees from Servier, Canada, outside of the current work. The remaining authors have nothing to disclose.

## Supporting information


**Table S1** Incidence Rates of All‐Cause Hospitalization According to Psychosocial Factors
**Table S2** Relation between Psychosocial Factors and First All‐Cause Hospitalization
**Figure S1** Flow Diagram of Patient Selection
**Figure S2** Cumulative incidence of all‐cause hospitalization according to psychosocial factors

## Data Availability

The patient informed consent forms, as approved by the responsible ethics committee (Ethikkommission Nordwest‐ und Zentralschweiz), do not allow the data to be made publicly available. The participants signed a consent form, which states that their data, containing personal and medical information, are exclusively available for research institutions in an anonymized form. Researchers interested in obtaining the data for research purposes can contact the Swiss‐AF scientific lead. Contact information is provided on the Swiss‐AF website (http://www.swissaf.ch/contact.htm). Authorization of the responsible ethics committee is mandatory before the requested data can be transferred to external research institutions.
