# Supplementary material for: Association of psychosocial factors with all‐cause hospitalizations in patients with atrial fibrillation
Source: Clin Cardiol. 2020 Nov 10;44(1):51–7. doi: 10.1002/clc.23503 (PMC7803348; doi:10.1002/clc.23503)
Supplement: Supplementary file 1 — Table S1 Incidence Rates of All‐Cause Hospitalization According to Psychosocial Factors Table S2 Relation between Psychosocial Factors and First All‐Cause Hospitalization Figure S1 Flow Diagram of Patient Selection Figure S2 Cumulative incidence of all‐cause hospitalization according to psychosocial factors [file CLC-44-51-s001.docx]

***Association of Psychosocial Factors With All-Cause Hospitalizations***

***in Patients With Atrial Fibrillation***

Pascal Meyre, Anne Springer, Stefanie Aeschbacher, Steffen Blum, Nicolas Rodondi, Jürg H. Beer, Marcello Di Valentino, Christine Meyer-Zürn, Manuel Blum, Peter Ammann, Rebecca Mathys, Leo H. Bonati, Christian Sticherling, Matthias Schwenkglenks, Michael Kühne, David Conen, and Stefan Osswald, on behalf of the Swiss-AF Investigators

**SUPPLEMENT**

**Table S1** Incidence Rates of All-Cause Hospitalization According to Psychosocial Factors

**Table S2** Relation between Psychosocial Factors and First All-Cause Hospitalization

**Figure S1** Flow Diagram of Patient Selection

**Figure S2** Cumulative incidence of all-cause hospitalization according to psychosocial factors

**Swiss-AF Investigators**

University Hospital Basel/Basel University: Stefanie Aeschbacher, Steffen Blum, Leo Bonati, Peter Hämmerle, Philipp Krisai, Christine Meyer-Zürn, Pascal Meyre, Andreas U. Monsch, Christian Müller, Christiane Pudenz, Philipp Reddiess, Javier Ruperti Repilado, Aleksandra Schweizer, Anne Springer, Fabienne Steiner, Christian Sticherling, Thomas Szucs, Gian Voellmin, Leon Zwimpfer. Local Principal Investigator: Michael Kühne; Principal Investigators; Stefan Osswald, David Conen.

University Hospital Bern: Faculty: Drahomir Aujesky, Urs Fischer, Juerg Fuhrer, Laurent Roten, Simon Jung, Heinrich Mattle; Research fellows: Luise Adam, Carole Elodie Aubert, Martin Feller, Claudio Schneider, Axel Loewe, Elisavet Moutzouri; Study nurses: Tanja Flückiger, Cindy Groen, Nathalie Schwab. Local Principal Investigator: Nicolas Rodondi.

Stadtspital Triemli Zurich: Christopher Beynon, Roger Dillier, Franz Eberli, Christine Franzini, Isabel Juchli, Claudia Liedtke, Jacqueline Nadler, Thayze Obst, Noreen Tynan, Xiaoye Schneider, Katrin Studerus, Dominik Weishaupt. Local Principal Investigator: Andreas Müller.

Kantonspital Baden: Simone Fontana, Silke Kuest, Karin Scheuch, Denise Hischier, Nicole Bonetti, Corina Bello, Henriette Isberg, Alexandra Grau, Jonas Villinger, Mary-Monica Papaux, Eva Laube, Philipp Baumgartner, Mark Filipovic, Marcel Frick, Stefanie Leuenberger. Local Principal Investigator: Jürg H. Beer.

Cardiocentro Lugano: Angelo Auricchio, Adriana Anesini, Cristina Camporini, Giulio Conte, Maria Luce Caputo, Francois Regoli, Tiziano Moccetti. Local Principal Investigator: Tiziano Moccetti.

Kantonsspital St. Gallen: Roman Brenner, David Altmann, Manuela Forrer, Michaela Gemperle. Local Principal Investigator: Peter Ammann.

Hôpital Cantonal Fribourg: Mathieu Firmann, Sandrine Foucras. Local Principal Investigator: Daniel Hayoz.

Luzerner Kantonsspital: Benjamin Berte, Andrea Kaeppeli, Myriam Roth, Brigitta Mehmann, Markus Pfeiffer, Ian Russi, Kai Schmidt, Vanessa Weberndoerfer, Mabelle Young, Melanie Zbinden; Local Principal Investigator: Richard Kobza.

Ente Ospedaliero Cantonale Lugano: Luisa Vicari, Jane Frangi-Kultalahti, Tatiana Terrot. Local Principal Investigator: Giorgio Moschovitis.

University Hospital Geneva: Georg Ehret, Hervé Gallet, Elise Guillermet, Francois Lazeyras, Karl-Olof Lovblad, Patrick Perret, Cheryl Teres. Local Principal Investigator: Dipen Shah.

University Hospital Lausanne: Nathalie Lauriers, Marie Méan, Sandrine Salzmann. Local Principal Investigator: Jürg Schläpfer.

Bürgerspital Solothurn: Nisha Arenja, Andrea Grêt, Sandra Vitelli. Local Principal Investigator: Frank-Peter Stephan.

Ente Ospedaliero Cantonale Bellinzona: Jane Frangi, Augusto Gallino. Local Principal Investigator: Marcello Di Valentino.

St. Anna Spital Luzern: Renate Schoenenberger-Berzins.

University of Zurich/University Hospital Zurich: Fabienne Witassek, Matthias Schwenkglenks, Christoph Stippich.

Medical Image Analysis Center AG Basel: Ernst-Wilhelm Radue, Tim Sinnecker, Jens Würfel.

Clinical Trial Unit Basel: Pascal Benkert, Thomas Fabbro, Patrick Simon, Michael Coslovsky.

Schiller AG Baar: Ramun Schmid.

**Table S1 Incidence Rates of All-Cause Hospitalization According to Psychosocial Factors**

|  | **All-cause hospitalization** | |
| --- | --- | --- |
| **Psychosocial factors** | **Events/ No. of patients** | **Incidence^a^** |
| **Marital status** |  |  |
| Married | 571/1597 | 17.7 (16.3-19.2) |
| Single | 69/156 | 23.5 (18.6-29.8) |
| Divorced | 119/289 | 21.8 (18.2-26.1) |
| Widowed | 132/336 | 22.3 (18.8-26.5) |
| **Education** |  |  |
| College, or university | 313/916 | 17.1 (15.3-19.1) |
| Secondary | 469/1181 | 20.5 (18.8-22.5) |
| Primary or less | 109/281 | 20.5 (17.0-24.7) |
| **Depression** |  |  |
| No | 835/2266 | 18.7 (17.5-20.0) |
| Yes | 56/112 | 29.1 (22.4-37.9) |
| **Health perception** |  |  |
| 1. Quartile (100-86) | 139/502 | 12.9 (10.9-15.2) |
| 2. Quartile (85-81) | 59/171 | 16.4 (12.7-21.2) |
| 3. Quartile (80-61) | 371/1047 | 17.5 (15.8-19.4) |
| 4. Quartile (<61) | 322/658 | 29.4 (26.3-32.7) |
| ^a^ Incidence per 100 patient-years of follow-up (95% CI) | | |

**Table S2 Relation between Psychosocial Factors and First All-Cause Hospitalization**

|  | **First all-cause hospitalization** | | | | | | | |
| --- | --- | --- | --- | --- | --- | --- | --- | --- |
| **Variables** | **No. of events*** | **No.**  **at risk** | **Unadjusted**  **HR (95% CI)** | **P value** | **Adjusted**  **HR (95% CI)**† | **P value** | **Combined adjusted**  **HR (95% CI)**‡ | **P value** |
| **Marital status** |  |  |  |  |  |  |  |  |
| Married | 571 | 1597 | 1 [Reference] |  | 1 [Reference] |  | 1 [Reference] |  |
| Single | 69 | 156 | 1.31 (1.02-1.68) | 0.034 | 1.37 (1.06-1.77) | 0.015 | 1.35 (1.05-1.74) | 0.021 |
| Divorced | 119 | 289 | 1.22 (1.00-1.49) | 0.047 | 1.25 (1.02-1.53) | 0.030 | 1.23 (1.00-1.50) | 0.046 |
| Widowed | 132 | 336 | 1.23 (1.02-1.50) | 0.031 | 1.05 (0.86-1.29) | 0.61 | 1.03 (0.84-1.27) | 0.74 |
| **Education** |  |  |  |  |  |  |  |  |
| College, or university | 313 | 916 | 1 [Reference] |  | 1 [Reference] |  | 1 [Reference] |  |
| Secondary | 469 | 1181 | 1.20 (1.04-1.38) | 0.013 | 1.14 (0.99-1.32) | 0.08 | 1.16 (1.00-1.35) | 0.05 |
| Primary or less | 109 | 281 | 1.19 (0.96-1.48) | 0.12 | 1.11 (0.88-1.40) | 0.39 | 1.10 (0.87-1.39) | 0.43 |
| **Depression** |  |  |  |  |  |  |  |  |
| No | 835 | 2266 | 1 [Reference] |  | 1 [Reference] |  | 1 [Reference] |  |
| Yes | 56 | 112 | 1.54 (1.17-2.01) | 0.002 | 1.27 (0.96-1.68) | 0.09 | 1.15 (0.87-1.52) | 0.34 |
| **Health perception** |  |  |  |  |  |  |  |  |
| 1. Quartile (100-86) | 139 | 502 | 1 [Reference] |  | 1 [Reference] |  | 1 [Reference] |  |
| 2. Quartile (85-81) | 59 | 171 | 1.28 (0.94-1.73) | 0.12 | 1.22 (0.90-1.65) | 0.21 | 1.22 (0.90-1.65) | 0.21 |
| 3. Quartile (80-61) | 371 | 1047 | 1.35 (1.11-1.65) | 0.002 | 1.13 (0.93-1.38) | 0.23 | 1.13 (0.92-1.38) | 0.24 |
| 4. Quartile (<61) | 322 | 658 | 2.22 (1.82-2.71) | <0.001 | 1.53 (1.24-1.90) | <0.001 | 1.49 (1.21-1.85) | <0.001 |
| Data are presented as hazard ratios (HR) with 95% confidence intervals (CI).  * Events included first hospital admission.  † Models were adjusted for age, sex, body mass index, hypertension, diabetes, coronary heart disease, prior stroke/TIA, heart failure, peripheral vascular disease, renal failure, cancer, and previous falls.  ‡ Model was combined and adjusted for age, sex, body mass index, hypertension, diabetes, coronary heart disease, prior stroke/TIA, heart failure, peripheral vascular disease, renal failure, cancer, and previous falls. | | | | | | | | |

**Figure S1 Flow Diagram of Patient Selection**

**Figure S2 Cumulative incidence of all-cause hospitalization according to psychosocial factors**
